# Acceptance and self-reported continued use of AI virtual avatars: an extended UTAUT model with the moderating role of use frequency

**DOI:** 10.3389/fpsyg.2026.1957852

**Published:** 2026-10-01

**Authors:** Changsheng Liu, Miaohui Wu, Ge Wu

**Affiliations:** 1 School of Art and Design, Guangdong University of Technology, Guangzhou, China; 2 School of Philosophy, Huazhong University of Science and Technology, Wuhan, China

**Keywords:** AI virtual avatar, artificial intelligence, behavioral intention, continued-use tendency, facilitating conditions, PLS-SEM, use frequency, UTAUT

## Abstract

AI-driven virtual avatars are increasingly encountered in livestreaming and digital-content settings. This cross-sectional study examined an extended Unified Theory of Acceptance and Use of Technology (UTAUT) model using survey data from 165 Chinese adults reporting prior exposure to AI virtual-avatar livestreams or videos. PLS-SEM showed that effort expectancy and social influence were positively associated with behavioral intention, while performance expectancy was not statistically significant (*β* = 0.107, *p* = 0.176). Behavioral intention and facilitating conditions were positively associated with self-reported continued-use tendency. Use frequency had no significant direct association with this outcome, but positively moderated its association with facilitating conditions and negatively moderated its association with behavioral intention. The model explained 68.7% of the variance in intention and 71.0% in continued-use tendency. PLSpredict yielded positive *Q*^2^predict values and lower RMSE than the linear-model benchmark for all six endogenous indicators. The findings highlight frequency-related differences in continued-use associations and are interpreted as exploratory given the cross-sectional design and overlap among several measures.

## 1 Introduction

### 1.1 AI virtual avatars as a media-use context

Generative models, multimodal synthesis, and real-time animation have expanded the range of digital personas encountered in contemporary media. In this study, an AI virtual avatar is defined as an anthropomorphic digital character whose content or interaction is generated or substantially supported by artificial intelligence. This umbrella term is distinct from role-based labels: a virtual idol primarily denotes an entertainment identity, a virtual influencer a social-media or marketing role, and an AI virtual anchor a presenting role in news or livestream commerce. These categories overlap only when the persona is AI-driven; the empirical scope here is limited to AI-driven virtual-avatar content encountered in livestreams or videos (Miao et al., 2022; Yu et al., 2023; Mouritzen et al., 2024; Audrezet et al., 2025).

Research on AI adoption generally finds that perceived usefulness or performance expectancy, effort expectancy, trust, and contextual support are associated with users’ intentions and use-related responses (Kelly et al., 2023; Menon and Shilpa, 2023; Strzelecki, 2023). AI virtual avatars add an anthropomorphic and socially embedded media context in which interaction, community discussion, and platform accessibility may be especially salient. Studies of AI and virtual influencers likewise show that anthropomorphism, perceived authenticity, and social presence can shape audience responses (Thomas and Fowler, 2021; Sands et al., 2022; Lou et al., 2023). Applying UTAUT to this context therefore requires attention not only to the presence of its established predictors, but also to the conditions under which their relative importance differs.

The study focuses on a Chinese, younger-adult-heavy sample because AI virtual-avatar content is widely circulated through livestream and interest-community platforms in this setting. Prior work suggests that social norms and perceived human-likeness can matter in virtual-idol and virtual-influencer engagement, while AI virtual anchors have become salient in livestream-commerce research (Arsenyan and Mirowska, 2021; Du et al., 2025; Stein et al., 2024; Zhu et al., 2025). These contextual observations motivate the study but do not imply that all Chinese or younger users share the same orientations. Figure 1 illustrates this technological evolution.

**Figure 1:**
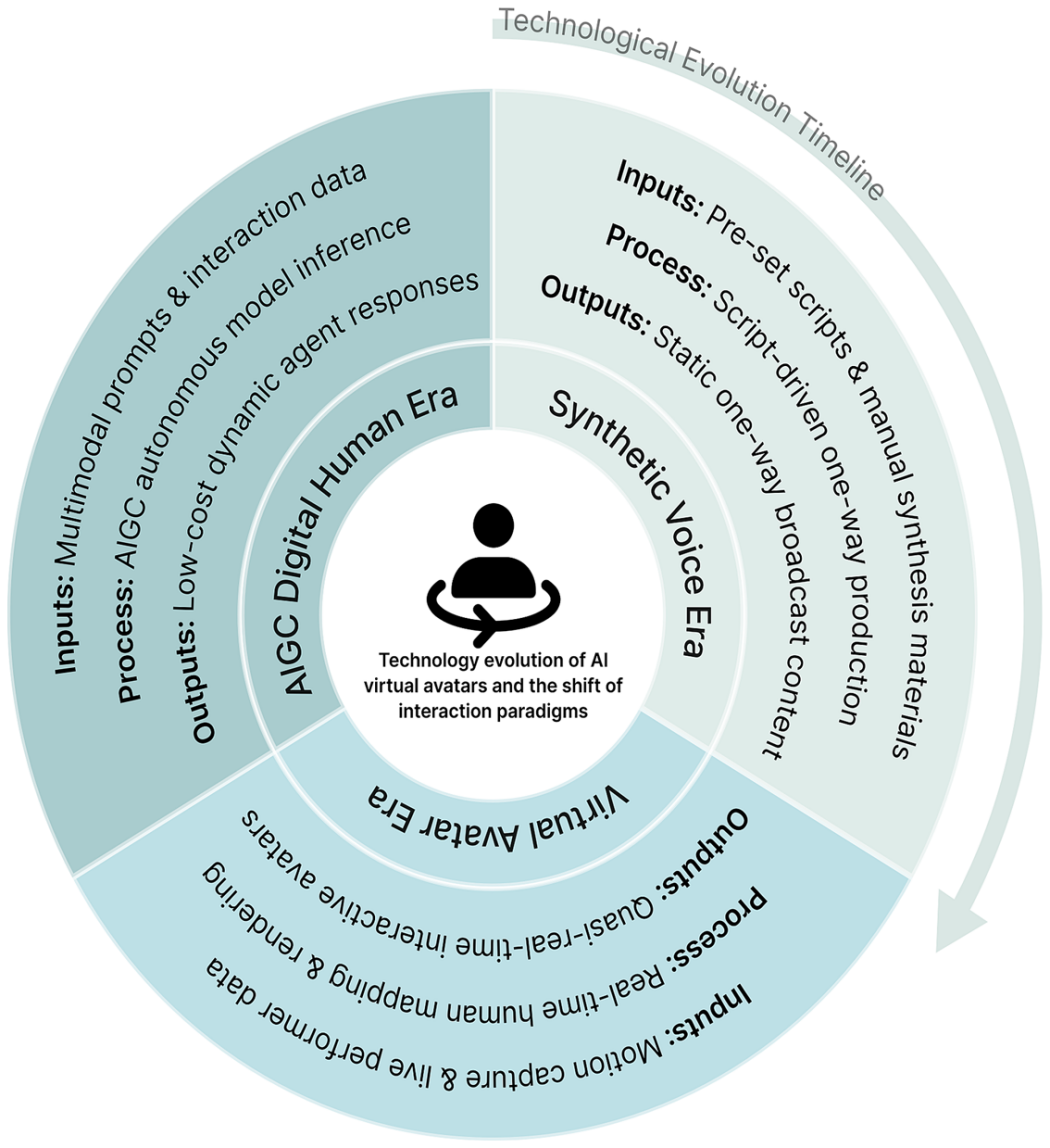
AI Virtual avatar technology evolution and the shift in interaction Paradigms/Source: Authors’ illustration.

### 1.2 Research gap, questions, and contribution

UTAUT identifies performance expectancy, effort expectancy, and social influence as antecedents of behavioral intention, and behavioral intention and facilitating conditions as predictors of technology use (Venkatesh et al., 2003). Post-adoption research distinguishes intention, facilitating conditions, experience, and repeated use, while consumer-oriented UTAUT extensions additionally incorporate habit (Bhattacherjee, 2001; Kim and Malhotra, 2005; Venkatesh et al., 2008, 2012). However, research on AI and avatar adoption has more often tested direct associations than examined whether the intention-use and facilitating conditions-use associations vary with users’ prior exposure frequency. This boundary-condition question is particularly relevant in voluntary media use, where respondents differ substantially in familiarity with platforms and content.

Use frequency, habit, and the outcome measured in this study refer to different aspects of engagement. Use frequency is an ordered, single-item report of how often respondents watched AI virtual-avatar livestreams or videos during a typical month. Habit concerns learned automatic responding, which requires measures beyond frequency (Verplanken and Orbell, 2003). The outcome originally labeled Use Behavior (UB) combines recommendation, intended regular continuation, and perceived growth in interaction time. Throughout the manuscript and figures, UB denotes self-reported continued-use tendency; the code is retained for continuity with the analysis outputs. This composite captures reported orientations toward engagement, rather than directly recorded behavior.

Accordingly, the study addresses three questions: (RQ1) How are the core UTAUT predictors associated with behavioral intention and self-reported continued-use tendency in the AI virtual-avatar context? (RQ2) Do the associations of behavioral intention and facilitating conditions with continued-use tendency differ across levels of self-reported use frequency? (RQ3) Does the model demonstrate out-of-sample predictive performance for the endogenous indicators?

The study extends UTAUT to AI virtual-avatar media engagement by examining use frequency as a boundary condition on the intention–continuation and facilitating-conditions–continuation associations. It provides contextual evidence on how reported frequency relates to these association patterns. Questions of autonomy, reflective use, and digital inclusion motivate subsequent research with direct measures and longitudinal designs.

### 1.3 Literature review and hypothesis development

#### 1.3.1 UTAUT and AI-mediated consumer engagement

UTAUT integrates established technology-acceptance perspectives, including the usefulness and ease-of-use logic of the Technology Acceptance Model, and proposes that performance expectancy, effort expectancy, and social influence predict behavioral intention, whereas behavioral intention and facilitating conditions predict use (Davis, 1989; Venkatesh et al., 2003). UTAUT2 extends this logic to voluntary consumer settings and distinguishes habit from experience-related moderators (Venkatesh et al., 2012). This distinction is important here because the present study measures exposure frequency but not behavioral automaticity.

Across AI applications, performance and effort expectations remain common correlates of acceptance, but their relative strength varies with context (Kelly et al., 2023). Research on ChatGPT, for example, supports the usefulness of UTAUT while also highlighting interactivity, trust, and institutional context (Menon and Shilpa, 2023; Strzelecki, 2023). These findings support retaining the UTAUT core while avoiding the assumption that performance expectancy must always be the strongest antecedent.

AI virtual avatars combine technology use with mediated social presence. Work on avatar marketing and virtual influencers suggests that anthropomorphism, perceived similarity, authenticity, credibility, and role design shape audience responses (Arsenyan and Mirowska, 2021; Miao et al., 2022; Lou et al., 2023; Stein et al., 2024; Mouritzen et al., 2024). The present model does not measure parasocial interaction or trust; these studies instead provide context for adapting UTAUT wording to an anthropomorphic media object. Related research on museum digital humans shows that information richness, aesthetic experience, perceived usefulness, ease of use, and flow can jointly shape usage intention (Mo et al., 2026).

Social influence refers to the perceived importance of others’ views and social environments for one’s technology-related choices (Venkatesh et al., 2003). Community discussion and shared-interest groups are visible features of virtual-avatar consumption, and prior evidence indicates that collectivist orientation and virtual-idol characteristics can be associated with consumption intentions (Du et al., 2025). The analysis therefore examines social influence as an antecedent of behavioral intention without interpreting it as coercion.

Facilitating conditions refer to perceived access to resources, knowledge, and technical support required for use. In AI-mediated content settings, platform availability, device capability, and network stability can reduce practical barriers. Studies in other AI and software-use contexts likewise show that facilitating conditions can be an important correlate of use-related outcomes (Li and Wei, 2025; Yuan et al., 2023). An extended UTAUT study of family caregivers likewise linked facilitating conditions, performance expectancy, trust, and social influence to AI nursing acceptance (Ye et al., 2025).

#### 1.3.2 Use frequency as a boundary condition

The intention-behavior relationship is not invariant across all users and situations. Repetition in stable contexts can contribute to automatic responding, but past frequency is not itself a direct measure of habit (Ouellette and Wood, 1998; Verplanken and Orbell, 2003). Information-systems research likewise shows that habit can limit the predictive role of intention in continued use, while longitudinal work distinguishes this process from cross-sectional frequency differences (Kim and Malhotra, 2005; Limayem et al., 2007; Gardner et al., 2020; Söllner et al., 2024).

In the present cross-sectional design, use frequency is treated as an observed exposure characteristic. A negative frequency-by-intention interaction would indicate a weaker association between intention and continued-use tendency among respondents reporting more frequent exposure. Establishing a transition toward habitual use would require repeated observations and a direct measure of automaticity.

Conversely, frequent users may encounter platform, device, and network requirements more regularly, making facilitating conditions more closely associated with their continued-use responses. Prior work also shows that behavioral intention and facilitating conditions relate differently to alternative conceptualizations of system use (Venkatesh et al., 2008). A positive frequency-by-facilitating-conditions interaction would therefore indicate a stronger conditional association at higher reported frequency, not a causal increase in dependence.

Frequency may also show a direct association with continued-use tendency because past engagement and future continuation can covary. Testing this direct path while simultaneously estimating the interaction terms helps distinguish a general frequency association from frequency-contingent changes in the two focal relationships (see Figure 2).

**Figure 2:**
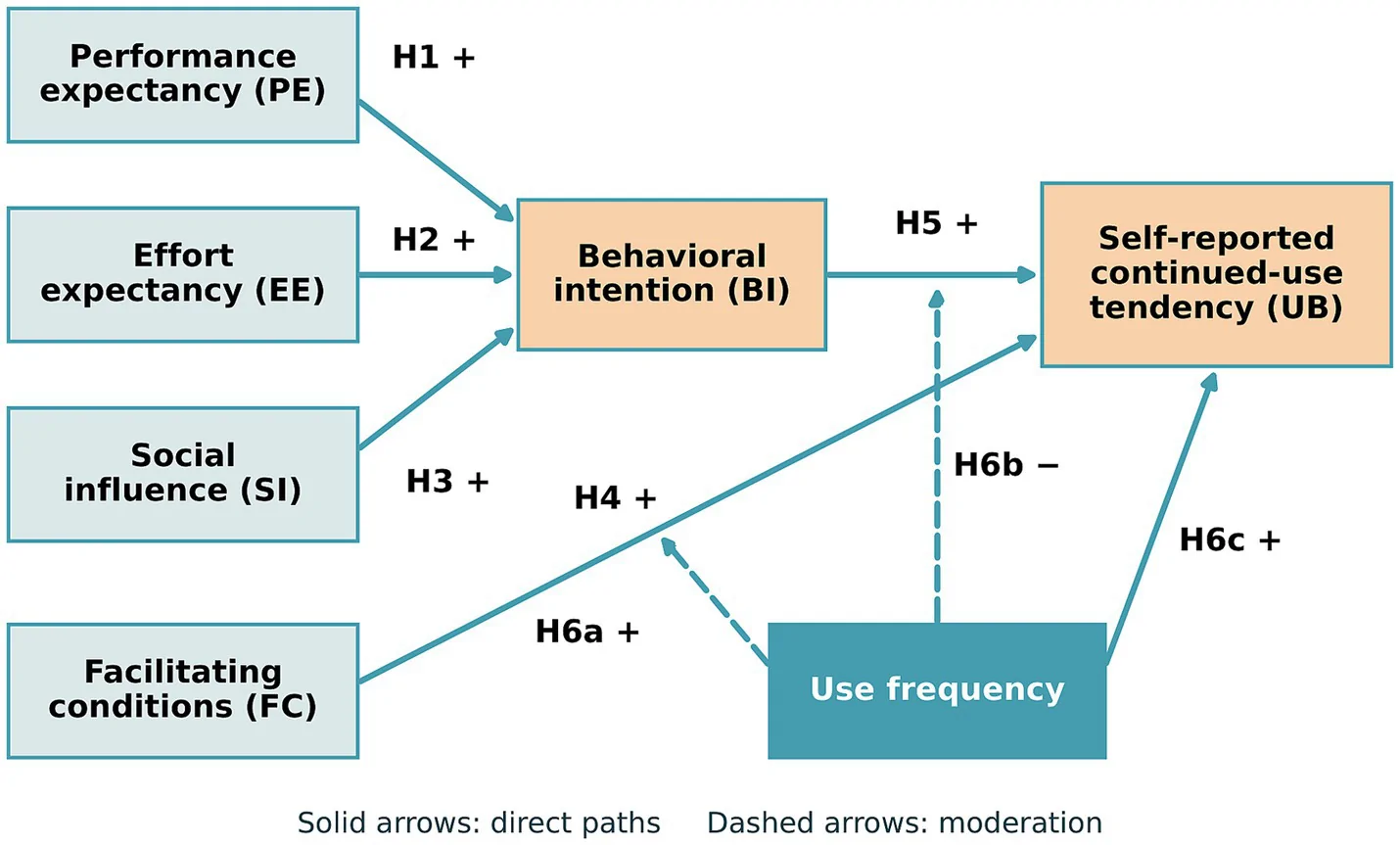
Extended UTAUT research model. Solid arrows denote direct paths, including H6c (Use Frequency → Self-Reported Continued-Use Tendency); dashed arrows denote the two moderation hypotheses. Signs indicate hypothesized directions, not significance. Source: authors’ illustration.

This formulation positions frequency as a measured moderator, habit as an unmeasured theoretical construct, and continued-use tendency as a self-reported outcome. The hypotheses below follow this deliberately restricted interpretation.

#### 1.3.3 Research hypotheses

Consistent with UTAUT and the contextual evidence reviewed above, the following hypotheses are proposed.

Performance expectancy reflects perceived benefits obtained from engaging with AI virtual avatars.

*H1*: Performance Expectancy (PE) is positively associated with Behavioral Intention (BI).

Effort expectancy reflects perceived ease and low interaction burden.

*H2*: Effort Expectancy (EE) is positively associated with Behavioral Intention (BI).

Social influence reflects perceived influence from relevant others and communities.

*H3*: Social Influence (SI) is positively associated with Behavioral Intention (BI).

Facilitating conditions reflect perceived platform, device, network, and access support.

*H4*: Facilitating Conditions (FC) are positively associated with Self-Reported Continued-Use Tendency (UB).

Behavioral intention represents respondents’ plans to continue engaging.

*H5*: Behavioral Intention (BI) is positively associated with Self-Reported Continued-Use Tendency (UB).

*H6a*: Use Frequency positively moderates the association between Facilitating Conditions and Self-Reported Continued-Use Tendency.

*H6b*: Use Frequency negatively moderates the association between Behavioral Intention and Self-Reported Continued-Use Tendency.

*H6c*: Use Frequency is positively associated with Self-Reported Continued-Use Tendency.

Figure 2 summarizes the hypothesized direct and moderating relationships.

### 1.4 Research model

The model retains the UTAUT core paths and adds self-reported use frequency as a direct predictor of continued-use tendency (H6c), as well as a moderator of the FC–UB and BI–UB paths (H6a and H6b; Figure 2). The diagram presents the hypothesized paths, including paths that are not statistically supported in the results.

## 2 Materials and methods

### 2.1 Measurement and operational definitions

The questionnaire was administered in Chinese. The English renderings in Table 1 document the administered item content. PE, EE, SI, FC, and BI were informed by technology-acceptance research, and UB by continuance research (Davis, 1989; Bhattacherjee, 2001; Venkatesh et al., 2003, 2008, 2012). The exact sentences were developed by the research team for AI virtual-avatar livestream and video settings; they were not verbatim translations of a single validated instrument. Table 1 distinguishes theoretical sources from item authorship. The original response scale was 1 (strongly agree) to 7 (strongly disagree). In the analysis file used for this revision, every retained multi-item response was transformed as x′ = 8 − x, so higher analyzed values represent stronger endorsement. Monthly use frequency retained its ascending 1–4 coding. This coding description was checked against the indicator data embedded in the SmartPLS exports. The measurement specification was reviewed during revision using theoretical item content and measurement diagnostics, and all item decisions are documented transparently.

**Table 1:** Construct definitions, retained measurement items, theoretical basis, and item authorship.

Construct and definition	Code	Retained item	Theoretical basis and exact item authorship
Performance Expectancy (PE): Perceived qualities and benefits of AI-avatar interaction, including human-like communication, distinctiveness, and self-expression.	PE1	I believe that interacting with generative AI Virtual Avatar can provide me with a human-like communication experience.	Theoretical basis: Venkatesh et al. (2003) and Miao et al. (2022); exact wording: research team
Performance Expectancy (PE): Perceived qualities and benefits of AI-avatar interaction, including human-like communication, distinctiveness, and self-expression.	PE2	I think each virtual avatar has a distinct personality.	Theoretical basis: Venkatesh et al. (2003) and Miao et al. (2022); exact wording: research team
Performance Expectancy (PE): Perceived qualities and benefits of AI-avatar interaction, including human-like communication, distinctiveness, and self-expression.	PE3	I think virtual avatars can actively express themselves.	Theoretical basis: Venkatesh et al. (2003) and Miao et al. (2022); exact wording: research team
Effort Expectancy (EE): Perceived ease and low cognitive effort of interacting with AI virtual-avatar content.	EE1	I think the interaction is simple, and my bullet comments or comments can receive a response or acknowledgment from the virtual avatar.	Theoretical basis: Venkatesh et al. (2003); exact wording: research team
Effort Expectancy (EE): Perceived ease and low cognitive effort of interacting with AI virtual-avatar content.	EE2	The interaction is simple and straightforward, and I feel that I can influence the live-streaming content to some extent.	Theoretical basis: Venkatesh et al. (2003); exact wording: research team
Effort Expectancy (EE): Perceived ease and low cognitive effort of interacting with AI virtual-avatar content.	EE3	The virtual avatar’s live room provides timely feedback to my actions, such as comments and gifts.	Theoretical basis: Venkatesh et al. (2003); exact wording: research team
Effort Expectancy (EE): Perceived ease and low cognitive effort of interacting with AI virtual-avatar content.	EE4	Interacting with the virtual avatar gives me a sense of participation and does not impose excessive cognitive burden.	Theoretical basis: Venkatesh et al. (2003); exact wording: research team
Social Influence (SI): Perceived influence of relevant others and communities on viewing or interaction.	SI1	In my community, people generally like and discuss AI Virtual Avatar.	Theoretical basis: Venkatesh et al. (2003) and Du et al. (2025); exact wording: research team
Social Influence (SI): Perceived influence of relevant others and communities on viewing or interaction.	SI3	When watching, even if I am influenced by friends, relatives, or fans with shared interests, I still focus my attention on the live stream or video.	Theoretical basis: Venkatesh et al. (2003) and Du et al. (2025); exact wording: research team
Social Influence (SI): Perceived influence of relevant others and communities on viewing or interaction.	SI4	I try new AI Virtual Avatar content because of discussions and recommendations within the community.	Theoretical basis: Venkatesh et al. (2003) and Du et al. (2025); exact wording: research team
Facilitating Conditions (FC): Perceived availability of platform, device, network, and access support.	FC1	I can easily find and access the AI Virtual Avatar content I want to watch.	Theoretical basis: Venkatesh et al. (2003) and Li and Wei (2025); exact wording: research team
Facilitating Conditions (FC): Perceived availability of platform, device, network, and access support.	FC2	Even when there are no temporary updates, I can conveniently access related content about my preferred AI Virtual Avatar without excessive barriers.	Theoretical basis: Venkatesh et al. (2003) and Yuan et al. (2023); exact wording: research team
Facilitating Conditions (FC): Perceived availability of platform, device, network, and access support.	FC3	I hope to have sufficiently capable devices and a smooth network when watching the virtual avatar I like.	Theoretical basis: Venkatesh et al. (2003) and Li and Wei (2025); exact wording: research team
Behavioral Intention (BI): Plans and willingness to continue engaging with AI virtual-avatar content.	BI1	After watching a virtual avatar on one platform, I will still prioritize the original platform even if the content is available elsewhere.	Theoretical basis: Venkatesh et al. (2003) and Bhattacherjee (2001); exact wording: research team
Behavioral Intention (BI): Plans and willingness to continue engaging with AI virtual-avatar content.	BI2	I will continue watching virtual avatars on the same platform in the future.	Theoretical basis: Venkatesh et al. (2003) and Bhattacherjee (2001); exact wording: research team
Behavioral Intention (BI): Plans and willingness to continue engaging with AI virtual-avatar content.	BI3	If there are activities or new content related to virtual avatars I like, I will actively participate.	Theoretical basis: Venkatesh et al. (2003) and Bhattacherjee (2001); exact wording: research team
Self-Reported Continued-Use Tendency (UB): Self-reported tendency to recommend, continue regularly, and increase engagement; not objective platform-recorded behavior.	UB1	I am willing to recommend the virtual avatars I watch and the viewing platforms to others.	Theoretical basis: Bhattacherjee (2001) and Menon (2022a,b); exact wording: research team
Self-Reported Continued-Use Tendency (UB): Self-reported tendency to recommend, continue regularly, and increase engagement; not objective platform-recorded behavior.	UB2	I intend to continue watching the virtual avatars I like as a regular weekly habit.	Theoretical basis: Bhattacherjee (2001) and Menon (2022a,b); exact wording: research team
Self-Reported Continued-Use Tendency (UB): Self-reported tendency to recommend, continue regularly, and increase engagement; not objective platform-recorded behavior.	UB3	Compared with my initial contact, the total time I now spend interacting with this AI has increased substantially.	Theoretical basis: Bhattacherjee (2001) and Menon (2022a,b); exact wording: research team

### 2.2 Sample collection and analysis

A cross-sectional online questionnaire was programmed and distributed through Wenjuanxing. Recruitment messages were circulated through WeChat, Weibo, and communities related to AI-driven virtual-avatar content using online snowball recruitment. The survey introduction defined the focal technology and informed participants about voluntariness, anonymity, confidentiality, and the study purpose.

The research topic and questionnaire design process began in November 2025; no formal survey data were collected at that stage. The ethics application was submitted on 17 April 2026 and approved on 15 June 2026 (protocol GDUTXS20260194). The formal survey window ran from 15 June to 10 July 2026; the exported records were submitted between 15 June and 5 July 2026. The analytical sample was restricted *post hoc* during revision to adults reporting prior exposure. The original export contained 337 submissions. All 170 respondents who selected the ‘almost never’ frequency category lacked responses to the 21 original scale items. The other 167 records contained complete scale responses; excluding two additional respondents under 18 left 165 adults for analysis. Three other respondents under 18 were already among the 170 records without scale responses and were not counted twice. ‘Almost never’ is the recorded category and should not be equated with verified zero lifetime exposure.

We examined whether the original and restricted samples could be compared using the same structural model. A 337-case model could not be re-estimated from the available export because the 170 excluded frequency-category records contained no scale responses; imputing entire measurement profiles would require unsupported assumptions. The remaining two complete records were outside the adult target population. Figure 3 summarizes recruitment, response availability, and screening. Supplementary Table S4 reports the record-level accounting. The audit establishes record counts and data availability; possible selection bias remains a limitation. Separately, Supplementary Tables S5, S6 compare the retained-PE4 and deleted-PE4 specifications on the same 165 adults, addressing measurement sensitivity rather than sample-selection sensitivity.

**Figure 3:**
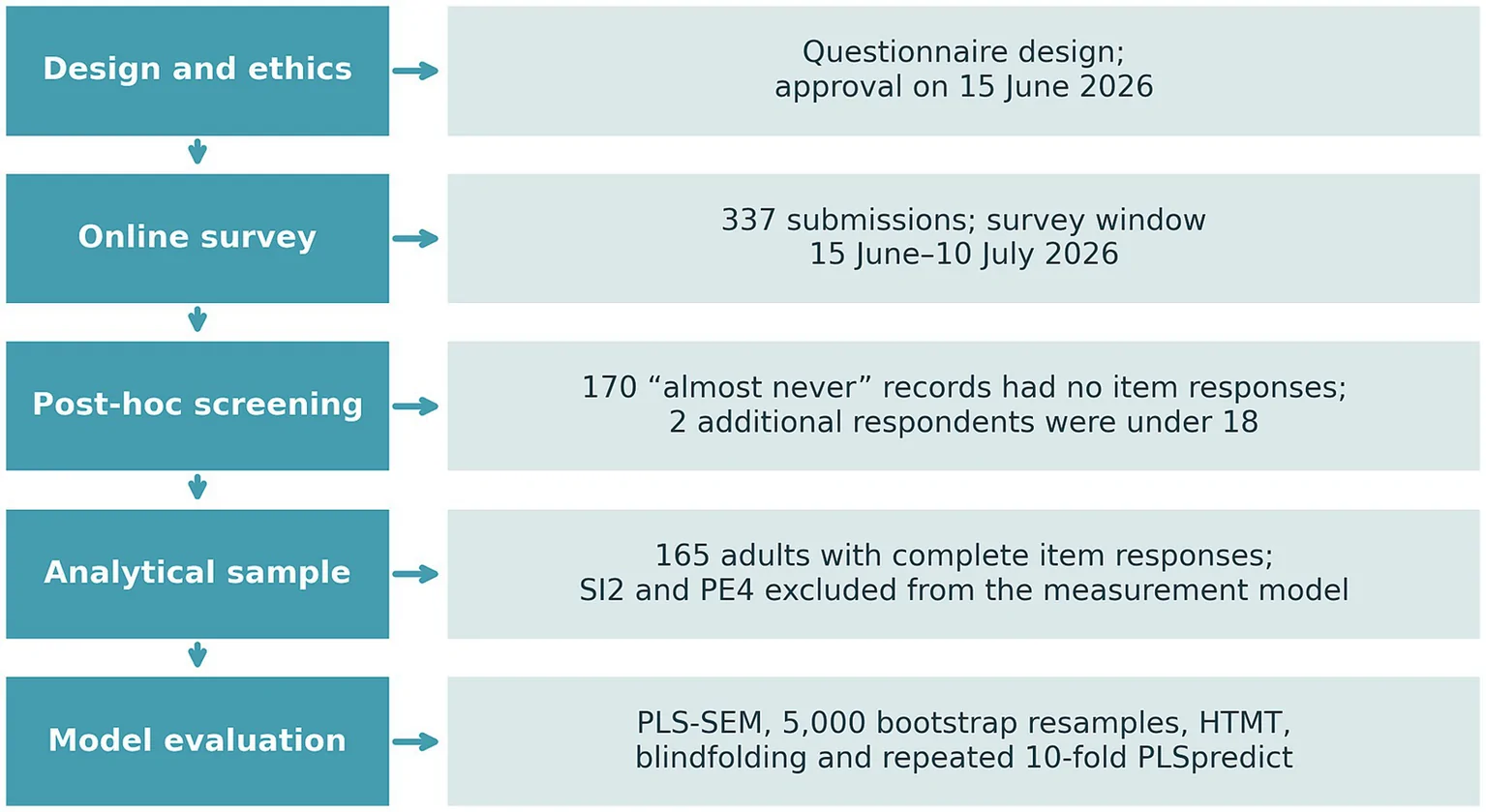
Recruitment, item-data availability, *post-hoc* sample restriction, and analysis procedure. The 170 records without scale responses and the two additional respondents under 18 were excluded sequentially. Source: authors’ illustration.

The final sample comprised 85 men (51.52%) and 80 women (48.48%). Respondents aged 19–25 years constituted 57.58% of the analytical sample. Monthly viewing-frequency categories were corrected to eliminate the previous overlap: 1–3 times, 4–6 times, 7–10 times, and more than 10 times. These categories were coded 1–4 in ascending order and entered as an ordered single-indicator moderator; higher values therefore represented greater reported frequency. For the interaction analysis, the ordered coding was treated as an approximately linear category score, so a one-unit change represents movement to the next frequency category rather than an exact increase in viewing occasions. A multiple-regression sensitivity analysis with five predictors, alpha = 0.05, *n* = 165, and 80% power indicated a minimum detectable effect of approximately *f*^2^ = 0.080 (Faul et al., 2009). Table 2 presents the complete profile of the filtered sample.

**Table 2:** Characteristics of the eligible analytical sample (*n* = 165).

Variable	Category	*n*	%
Gender	Male	85	51.52%
	Female	80	48.48%
Age	19–25 years	95	57.58%
	26–30 years	34	20.61%
	31–40 years	33	20.00%
	41–50 years	3	1.82%
Education	Junior high or below	3	1.82%
	High/vocational school	17	10.30%
	Junior college	37	22.42%
	Undergraduate	94	56.97%
	Graduate degree or above	14	8.48%
Monthly frequency	1–3 times	46	27.88%
	4–6 times	31	18.79%
	7–10 times	50	30.30%
	>10 times	38	23.03%

Analyses used SmartPLS 4 with reflective measurement blocks and path weighting. Internal consistency and convergent validity were assessed using item loadings, Cronbach’s alpha, rho_A, rho_c, and AVE (Hair et al., 2019, 2022). Discriminant validity was evaluated using HTMT point estimates with the conventional 0.85 and 0.90 reference thresholds and 95% percentile bootstrap confidence intervals (Henseler et al., 2015). Fornell–Larcker results and cross-loadings were retained as complementary diagnostics. The intervals describe pairwise uncertainty and were not adjusted for multiplicity or used to claim that the entire measurement model passed discriminant-validity testing. Structural assessment included VIF, *R*^2^, *f*^2^, and 5,000 bootstrap resamples with two-tailed tests, a 0.05 significance level, and the software’s fixed-seed option.

The moderation analysis used the two-stage approach, with frequency and the other construct scores standardized before creating the interaction products. Simple slopes were evaluated at −1 SD, the mean, and +1 SD of frequency, holding the other focal predictor at its mean. Product scaling was checked against the exported construct-score matrix. Blindfolding used omission distance 10. Separately, PLSpredict used 10 folds and 10 repetitions with a fixed seed to compare PLS-SEM prediction errors with linear-model (LM) and indicator-average benchmarks (Shmueli et al., 2019). The 1,650 prediction-error entries represent repeated predictions for 165 respondents, not independent additional participants.

## 3 Results

### 3.1 Reliability and validity assessment

SI2, which described ignoring surroundings and losing track of time, was removed in the first revision because it mainly captured absorption. SI3 was retained because it names friends, relatives, and shared-interest fans, but its attention-related clause remains a content-validity concern. The measurement specification was re-examined during revision. PE4 (‘I have a certain emotional connection with the AI Virtual Avatar I like’) was removed because it directly expresses emotional attachment, whereas the remaining PE items concern perceived avatar and interaction qualities. This modification followed theoretical reconsideration and assessment of the measurement properties. The revised specification was evaluated on the same sample and should be further validated in future studies. PE1–PE3, EE1–EE4, SI1/SI3/SI4, FC1–FC3, BI1–BI3, and UB1–UB3 were retained. The 19-item model was re-estimated on the same 165 respondents.

Retained outer loadings ranged from 0.802 to 0.888. Cronbach’s alpha ranged from 0.803 to 0.880, composite reliability (rho_c) from 0.884 to 0.917, and AVE from 0.718 to 0.750 (Table 3). These diagnostics indicate internal consistency and convergent validity, which are separate from evidence about construct distinctiveness.

**Table 3:** Reliability and convergent validity of the revised measurement model.

Construct	Item	Loading	alpha	rho_A	rho_c	AVE
PE	PE1	0.828	0.833	0.833	0.900	0.750
PE	PE2	0.880	0.833	0.833	0.900	0.750
PE	PE3	0.888	0.833	0.833	0.900	0.750
EE	EE1	0.872	0.880	0.886	0.917	0.735
EE	EE2	0.806	0.880	0.886	0.917	0.735
EE	EE3	0.866	0.880	0.886	0.917	0.735
EE	EE4	0.883	0.880	0.886	0.917	0.735
SI	SI1	0.838	0.831	0.833	0.899	0.748
SI	SI3	0.880	0.831	0.833	0.899	0.748
SI	SI4	0.876	0.831	0.833	0.899	0.748
FC	FC1	0.802	0.814	0.829	0.889	0.729
FC	FC2	0.874	0.814	0.829	0.889	0.729
FC	FC3	0.883	0.814	0.829	0.889	0.729
BI	BI1	0.830	0.813	0.815	0.889	0.728
BI	BI2	0.865	0.813	0.815	0.889	0.728
BI	BI3	0.865	0.813	0.815	0.889	0.728
UB	UB1	0.862	0.803	0.804	0.884	0.718
UB	UB2	0.842	0.803	0.804	0.884	0.718
UB	UB3	0.838	0.803	0.804	0.884	0.718

HTMT results for all 15 pairs of multi-item constructs are reported in Table 4. Nine point estimates exceeded 0.90, and seven 95% percentile bootstrap confidence intervals included 1.00. The closest pairs were BI–UB [0.980; 95% CI (0.915, 1.050)], SI–BI [0.977; (0.897, 1.056)], and SI–UB [0.968; (0.895, 1.035)]. Several construct pairs showed insufficient empirical separation under the HTMT criteria, particularly BI–UB and SI-related pairs. The complete 21-pair software list, including frequency, appears in Supplementary Table S1; the extended matrix including generated interaction terms appears in Supplementary Table S2. Frequency is a single-item measure and the interaction terms are generated variables, so their entries are reported for completeness rather than interpreted as validation of multi-item constructs.

**Table 4:** HTMT point estimates and 95% percentile bootstrap confidence intervals for all multi-item construct pairs.

Construct pair	HTMT	95% CI lower	95% CI upper
PE–EE	0.893	0.804	0.965
PE–SI	0.938	0.857	1.013
PE–FC	0.832	0.728	0.927
PE–BI	0.857	0.753	0.954
PE–UB	0.850	0.746	0.943
EE–SI	0.942	0.878	0.998
EE–FC	0.819	0.726	0.905
EE–BI	0.895	0.814	0.967
EE–UB	0.927	0.862	0.990
SI–FC	0.935	0.858	1.012
SI–BI	0.977	0.897	1.056
SI–UB	0.968	0.895	1.035
FC–BI	0.923	0.836	1.008
FC–UB	0.938	0.852	1.016
BI–UB	0.980	0.915	1.050

*n* = 165; 5,000 bootstrap resamples; two-sided percentile intervals. Point estimates above 0.90 indicate concerns under that criterion. Intervals are unadjusted pairwise intervals; including 1.00 does not prove that the constructs are identical.

Removing PE4 reduced the number of HTMT ratios above 0.90 from 13 to nine on the same sample (Supplementary Table S5). PE–SI fell from 0.979 to 0.938, but remained above 0.90. Ratios not involving PE were unchanged, including BI–UB, SI–BI, and SI–UB. The result is a partial improvement in the PE block, not a solution to the model-wide discriminant-validity problem.

The Fornell–Larcker comparisons met the conventional criterion (Supplementary Table S3), and each retained indicator had its highest loading on its assigned construct (Table 5). SI3 loaded 0.880 on SI, above its largest cross-loading of 0.696. These diagnostics do not override the HTMT concerns, because Fornell–Larcker and cross-loading checks can fail to detect insufficient discriminant validity (Henseler et al., 2015). The structural results are therefore presented with qualifications about overlapping constructs.

**Table 5:** Cross-loadings of retained indicators.

Item	BI	EE	FC	PE	SI	UB
BI1	0.830	0.604	0.607	0.562	0.664	0.644
BI2	0.865	0.645	0.624	0.595	0.663	0.701
BI3	0.865	0.696	0.701	0.651	0.731	0.684
EE1	0.676	0.872	0.664	0.678	0.711	0.666
EE2	0.580	0.806	0.561	0.606	0.597	0.635
EE3	0.625	0.866	0.551	0.652	0.705	0.649
EE4	0.717	0.883	0.622	0.685	0.744	0.722
FC1	0.578	0.499	0.802	0.513	0.588	0.547
FC2	0.708	0.621	0.874	0.635	0.686	0.700
FC3	0.641	0.659	0.883	0.607	0.697	0.698
PE1	0.622	0.651	0.615	0.828	0.641	0.596
PE2	0.629	0.666	0.577	0.880	0.689	0.630
PE3	0.583	0.670	0.598	0.888	0.694	0.580
SI1	0.667	0.753	0.653	0.692	0.838	0.676
SI3	0.696	0.663	0.663	0.692	0.880	0.689
SI4	0.723	0.681	0.689	0.641	0.876	0.687
UB1	0.685	0.650	0.664	0.635	0.673	0.862
UB2	0.684	0.677	0.614	0.604	0.695	0.842
UB3	0.647	0.658	0.667	0.530	0.642	0.838

### 3.2 Structural model assessment

Indicator VIF values ranged from 1.000 to 2.452 when the single-item frequency indicator was included; structural predictor VIF values ranged from 1.151 to 3.498.

Table 6 distinguishes measurement-model and structural-model VIF values. All were below 5 under the adopted descriptive benchmark (Becker et al., 2023).

**Table 6:** Measurement-model and structural-model variance inflation factors.

Level	Indicator/path	VIF
Measurement	BI1	1.673
Measurement	BI2	1.896
Measurement	BI3	1.844
Measurement	EE1	2.374
Measurement	EE2	1.851
Measurement	EE3	2.398
Measurement	EE4	2.452
Measurement	FC1	1.632
Measurement	FC2	1.887
Measurement	FC3	1.976
Measurement	Freq	1.000
Measurement	PE1	1.615
Measurement	PE2	2.238
Measurement	PE3	2.399
Measurement	SI1	1.744
Measurement	SI3	2.096
Measurement	SI4	2.000
Measurement	UB1	1.827
Measurement	UB2	1.712
Measurement	UB3	1.676
Structural	BI → UB	2.396
Structural	EE → BI	3.316
Structural	FC → UB	2.424
Structural	Freq → UB	1.151
Structural	PE → BI	2.949
Structural	SI → BI	3.498
Structural	Freq x FC → UB	2.186
Structural	Freq x BI → UB	2.120

Several structural VIF values exceeded 3. These collinearity diagnostics do not resolve the construct overlap identified by HTMT, and individual path coefficients require cautious interpretation.

### 3.3 Assessment of model explanatory power

The model explained 68.7% of the variance in Behavioral Intention and 71.0% in Self-Reported Continued-Use Tendency (Table 7).

**Table 7:** Coefficients of determination.

Construct	*R* ^2^	Adjusted *R*^2^
BI	0.687	0.681
UB	0.710	0.701

Adjusted *R*^2^ values were 0.681 for BI and 0.701 for UB. These in-sample values summarize variance explained in the observed composite scores.

### 3.4 Model fit assessment

The saturated-model SRMR was 0.053 and the estimated-model SRMR was 0.063 (Table 8). SRMR is reported as an auxiliary descriptive fit index and does not supersede the HTMT assessment.

**Table 8:** Model fit indices.

Index	Saturated model	Estimated model
SRMR	0.053	0.063
NFI	0.821	0.814

Model quality was evaluated jointly using reliability, validity, collinearity, explanatory power, and predictive performance (Henseler, 2021).

### 3.5 Predictive relevance and out-of-sample prediction

Blindfolding with omission distance 10 produced Stone–Geisser *Q*^2^ values of 0.494 for BI and 0.491 for UB. Separately, PLSpredict with 10 folds and 10 repetitions yielded positive *Q*^2^predict values for all six endogenous indicators (0.437–0.554). PLS-SEM RMSE was lower than LM RMSE for all six indicators, and PLS-SEM MAE was lower for five of six indicators (Table 9). Overall CVPAT showed lower loss than the indicator-average benchmark (*p* < 0.001) and the LM benchmark (average loss difference = −0.086, *t* = 2.401, *p* = 0.017). Construct-specific comparisons against LM were not significant for BI (*p* = 0.118) or UB (*p* = 0.088). The cross-validation results apply to questionnaire-response prediction within this sample; external validation in a new population remains necessary.

**Table 9:** Blindfolding and PLSpredict results.

Procedure/indicator	*Q*^2^ or *Q*^2^predict	PLS RMSE	LM RMSE	PLS MAE	LM MAE
Blindfolding: BI	0.494	—	—	—	—
Blindfolding: UB	0.491	—	—	—	—
PLSpredict: BI1	0.437	1.263	1.298	0.948	0.974
PLSpredict: BI2	0.457	1.179	1.227	0.883	0.894
PLSpredict: BI3	0.554	1.068	1.096	0.830	0.840
PLSpredict: UB1	0.506	1.145	1.214	0.913	0.968
PLSpredict: UB2	0.479	1.142	1.147	0.864	0.842
PLSpredict: UB3	0.472	1.149	1.182	0.854	0.904

### 3.6 Results analysis

Path estimates, two-tailed bootstrap tests, and 95% percentile confidence intervals are reported in Table 10 and Figure 4. Because the measurement model was revised after inspecting the same sample, the estimates are interpreted as exploratory associations. H1 and H6c were not supported; H2–H5, H6a, and H6b were statistically supported under the specified model.

**Table 10:** Structural paths and hypothesis tests.

Hypothesis/path	*β*	*t*	*p*	95% CI	Decision
H1: PE → BI	0.107	1.354	0.176	[−0.038, 0.274]	Not supported
H2: EE → BI	0.280	3.059	0.002	[0.101, 0.460]	Supported
H3: SI → BI	0.495	5.037	<0.001	[0.292, 0.678]	Supported
H4: FC → UB	0.397	5.009	<0.001	[0.248, 0.558]	Supported
H5: BI → UB	0.470	5.839	<0.001	[0.308, 0.622]	Supported
H6a: Freq × FC → UB	0.170	2.299	0.022	[0.021, 0.317]	Supported
H6b: Freq × BI → UB	−0.178	2.430	0.015	[−0.324, −0.037]	Supported
H6c: Freq → UB	0.031	0.689	0.491	[−0.055, 0.123]	Not supported

**Figure 4:**
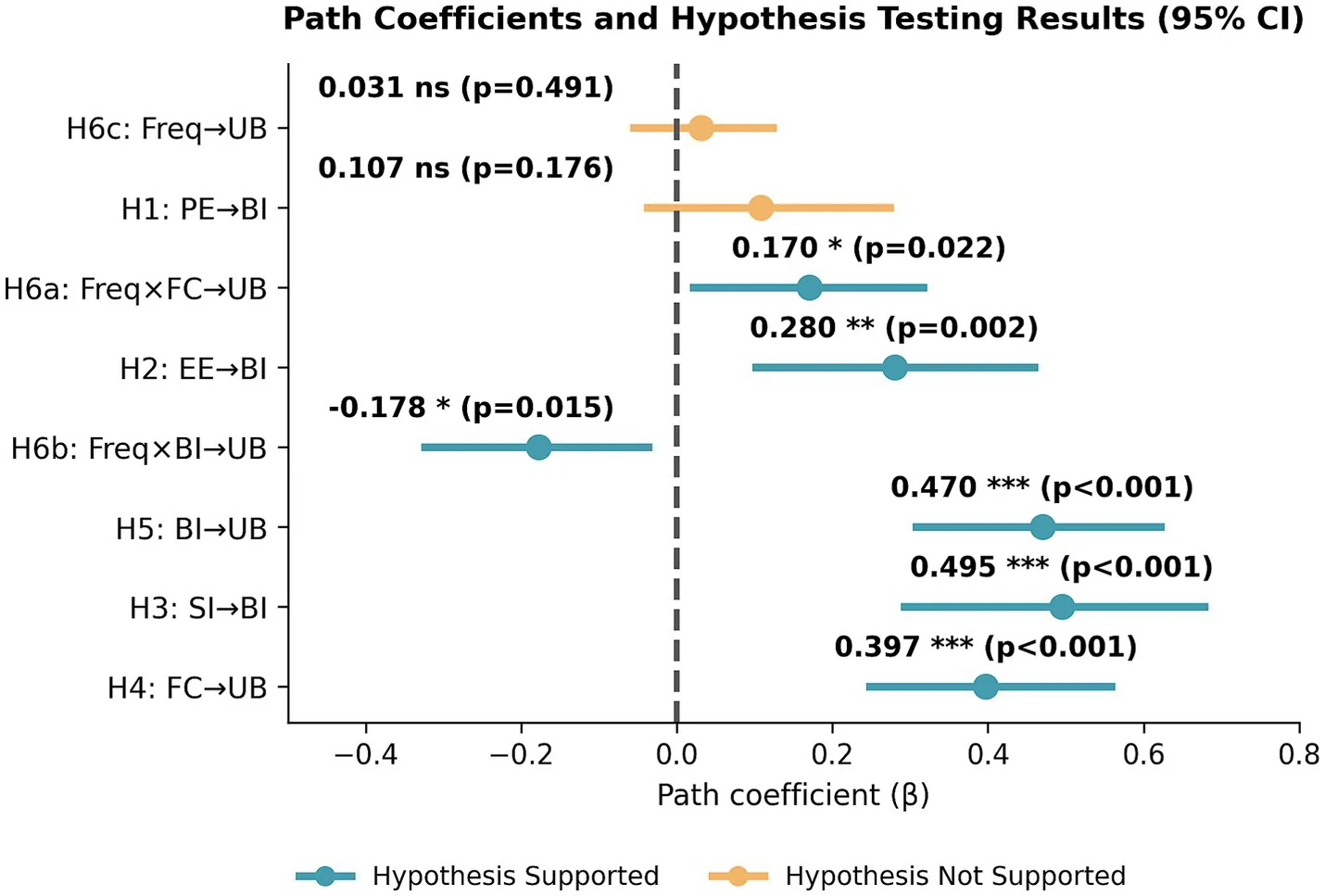
Path coefficients and 95% percentile bootstrap confidence intervals based on 5,000 resamples (*n* = 165). Teal indicates statistically supported hypotheses and orange indicates unsupported hypotheses (H1 and H6c). *p < 0.05, **p < 0.01, ***p < 0.001; ns, not significant. UB denotes self-reported continued-use tendency. Source: authors’ analysis.

### 3.7 Resulting model and hypothesis testing

The model explained 68.7% of BI and 71.0% of UB. Effect sizes (*f*^2^) were 0.012 for PE, 0.076 for EE, and 0.224 for SI in predicting BI; for UB, they were 0.318 for BI, 0.225 for FC, 0.003 for Frequency, 0.044 for Frequency × FC, and 0.053 for Frequency × BI. Using conventional benchmarks as descriptive guides, the interaction effects were small (Cohen, 1988). Their statistical significance does not remove the measurement limitations affecting the underlying constructs.

#### 3.7.1 Determinants of behavioral intention

Performance Expectancy had a positive but nonsignificant association with Behavioral Intention [β = 0.107, *t* = 1.354, *p* = 0.176; 95% CI (−0.038, 0.274)]; H1 was not supported.

Effort Expectancy was positively associated with Behavioral Intention [*β* = 0.280, *t* = 3.059, *p* = 0.002; 95% CI (0.101, 0.460)], supporting H2.

Social Influence was positively associated with Behavioral Intention [*β* = 0.495, *t* = 5.037, *p* < 0.001; 95% CI (0.292, 0.678)], supporting H3. Its point estimate was the largest of the three coefficients, but the high SI–BI and SI–EE HTMT values require cautious interpretation of this ordering as the relative importance of distinct psychological predictors.

Together, PE, EE, and SI accounted for 68.7% of the variance in BI. The change in H1 after removing PE4 is documented in Supplementary Table S6.

#### 3.7.2 Determinants of continued-use tendency

Behavioral Intention was positively associated with Self-Reported Continued-Use Tendency [*β* = 0.470, *t* = 5.839, *p* < 0.001; 95% CI (0.308, 0.622)], supporting H5 statistically. The BI–UB HTMT value of 0.980 indicates that shared item content may contribute to this association.

Facilitating Conditions were positively associated with Continued-Use Tendency [*β* = 0.397, *t* = 5.009, *p* < 0.001; 95% CI (0.248, 0.558)], supporting H4 under the specified model.

The direct association of Use Frequency with Continued-Use Tendency was small and nonsignificant [*β* = 0.031, *t* = 0.689, *p* = 0.491; 95% CI (−0.055, 0.123)]; H6c was not supported.

#### 3.7.3 Moderating effects of use frequency

The Frequency × Facilitating Conditions interaction was positive and significant [*β* = 0.170, *t* = 2.299, *p* = 0.022; 95% CI (0.021, 0.317)], supporting H6a.

The Frequency × Behavioral Intention interaction was negative and significant [*β* = −0.178, *t* = 2.430, *p* = 0.015; 95% CI (−0.324, −0.037)], supporting H6b.

These coefficients describe between-respondent differences in association: the FC–UB association was stronger, and the BI–UB association weaker, at higher reported use frequency.

The direct frequency coefficient did not provide evidence for H6c. The interaction estimates nevertheless indicate that the two focal associations differ across reported frequency levels in this sample.

Conditional slopes were calculated from the standardized main coefficients and the coefficients of the products of standardized scores at −1 SD, the mean, and +1 SD of frequency, with the other focal predictor held at its mean.

For the BI-UB association, the conditional slopes were 0.648 at low frequency, 0.470 at mean frequency, and 0.292 at high frequency. For the FC-UB association, the corresponding slopes were 0.227, 0.397, and 0.567.

Figure 5 displays these conditional associations using the exported score scaling and fitted intercept. The plotted slopes are descriptive values from one continuous interaction model; separate tests or confidence intervals for each conditional slope are not reported.

**Figure 5:**
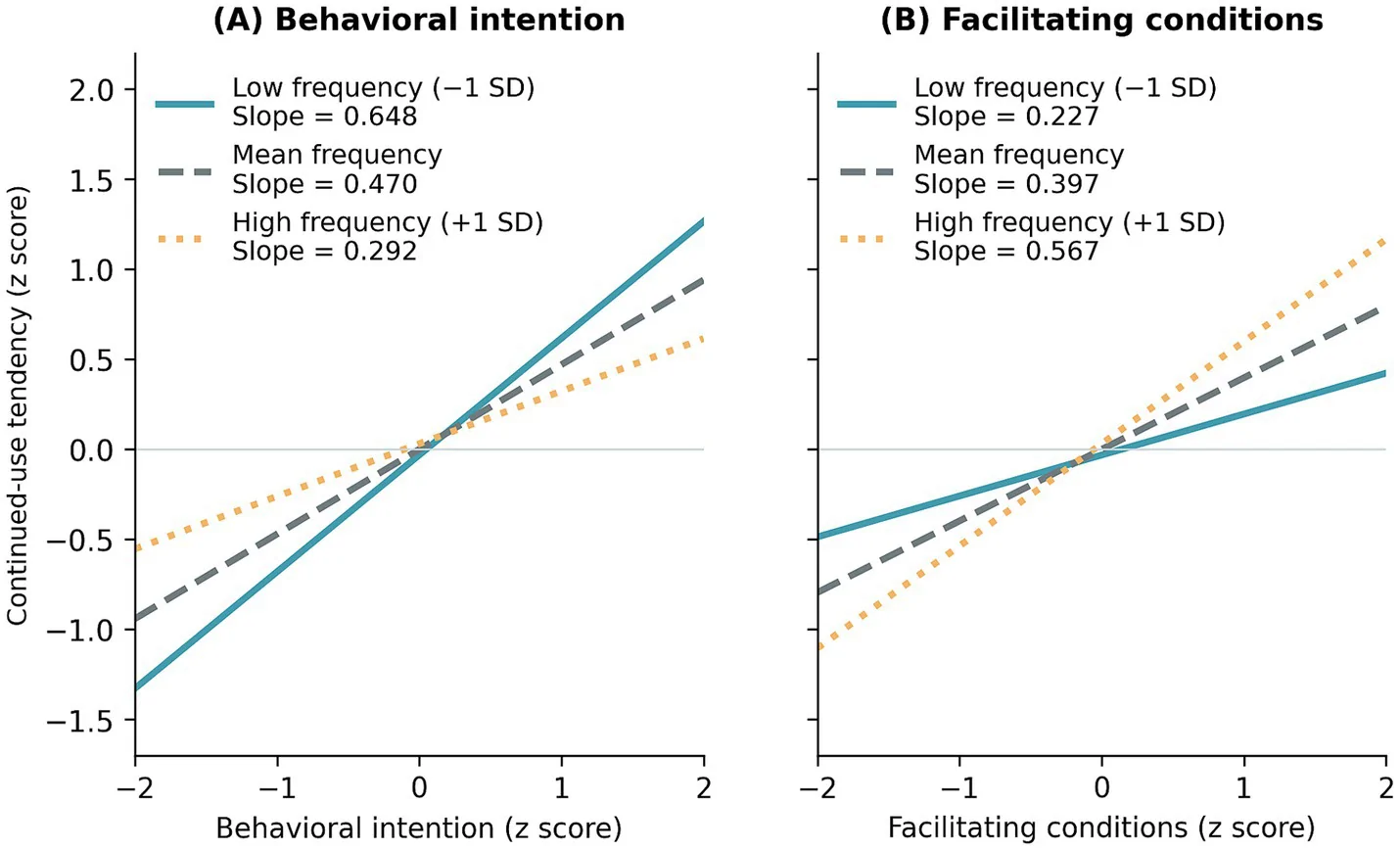
Conditional associations at low (−1 SD), mean, and high (+1 SD) use frequency. **(A)** BI–UB association with FC held at its mean. **(B)** FC–UB association with BI held at its mean. Curves depict fitted values and do not represent independent groups or longitudinal trajectories. UB denotes self-reported continued-use tendency. Source: authors’ analysis.

The interaction pattern indicates frequency-contingent differences in the relative associations of behavioral intention and facilitating conditions with continued-use tendency. Because frequency and continued-use tendency were measured at a single time point, the processes underlying these differences remain unresolved.

## 4 Discussion

In the 165-person analytical sample, the final specification statistically supported H2–H5 and the two moderation hypotheses. H1 and the direct frequency hypothesis H6c were not supported. The following interpretation considers these findings alongside the substantial HTMT overlap and the post-hoc measurement and sample decisions.

### 4.1 UTAUT relationships in AI virtual-avatar engagement

Effort Expectancy and Social Influence were positively associated with Behavioral Intention, whereas the Performance Expectancy coefficient was nonsignificant after PE4 was removed. This differs from the prior specification, in which the four-item PE coefficient was statistically significant. The comparison shows that the PE result is sensitive to how the construct is measured. Although SI had the largest coefficient among the predictors of BI, substantial measurement overlap makes comparisons of their relative contributions provisional. More differentiated measures are needed to evaluate the relative contributions proposed by UTAUT (Venkatesh et al., 2003).

SI1 and SI4 concern community discussion and recommendations, whereas SI3 also refers to sustained attention. This mixed content may partly account for the proximity of SI to other favorable engagement evaluations, although the present data do not identify its cause. Future work could separate perceived social expectations, exposure to recommendations, and absorption into distinct item sets before testing their relationships with intention.

### 4.2 Behavioral intention, facilitating conditions, and continued-use tendency

UB captures a broad continued-engagement orientation: willingness to recommend (UB1), intended regular viewing (UB2), and a perceived increase in interaction time (UB3). BI measures plans and willingness to continue engaging. In particular, BI and UB2 share prospective continuation wording, which may partly explain their HTMT value of 0.980. This result is consistent with overlap in measurement wording; it does not determine whether the underlying theoretical concepts are identical. H5 is therefore interpreted as an association between related engagement-oriented self-reports. A clearer test of intention–behavior correspondence would pair intention measures with subsequently recorded viewing or interaction behavior.

Facilitating Conditions were also associated with UB, but the FC–UB HTMT value of 0.938 limits claims about a separate resource-support mechanism. Shared administration and response format may also contribute to covariance, alongside substantive similarity; this possibility was not isolated by the present design. Follow-up studies should distinguish future plans from subsequently recorded viewing, interaction, and recommendation behavior, using appropriate consent procedures for platform records.

### 4.3 Use frequency as a boundary condition

The positive Frequency × Facilitating Conditions interaction and negative Frequency × Behavioral Intention interaction remained statistically significant after removing PE4. These estimates suggest stronger FC–UB and weaker BI–UB associations among respondents reporting higher frequency. Because the underlying measures overlap, they are exploratory patterns in the specified composites rather than confirmed differences between independently validated mechanisms.

The weaker BI-UB association and stronger FC-UB association at higher reported frequency may reflect greater platform familiarity, more stable access conditions, or differences in how experienced users evaluate continued engagement (Ouellette and Wood, 1998; Limayem et al., 2007; Gardner et al., 2020; Söllner et al., 2024).

Longitudinal research combining repeated surveys with behavioral logs could determine whether these between-person differences correspond to changes within individuals over time.

### 4.4 Contributions and implications

#### 4.4.1 Theoretical contributions

The study contributes to UTAUT research by examining two frequency-dependent associations in AI virtual-avatar engagement: a stronger FC–UB association and a weaker BI–UB association at higher reported frequency. These patterns identify questions for research on how practical support and stated intention relate to continued engagement at different exposure levels. Independent replication with more differentiated measures would assess the robustness of the proposed boundary condition.

The operational definitions distinguish monthly exposure frequency, prospective engagement plans (BI), and a broader continued-engagement orientation (UB). The observed overlap, especially around prospective continuation wording, identifies specific targets for improving AI-engagement questionnaires through cognitive interviews and independently piloted item sets.

The analysis combines in-sample explanation with internal out-of-sample prediction. Positive *Q*^2^predict values and lower PLS-SEM RMSE than LM across the endogenous indicators describe predictive performance within the cross-validation procedure (Shmueli et al., 2019). Prediction and construct distinctiveness address different aspects of model evaluation and are considered jointly in interpreting these findings.

#### 4.4.2 Practical and ethical implications

The associations motivate testing clear platform access, reliable performance, and usable interaction mechanisms as design features relevant to continued engagement. Evaluations could compare these features across levels of prior use frequency while measuring later behavior. Prospective field evaluations could test whether these design features improve later engagement and whether their effects differ by prior use frequency.

Understandable recommendation settings, accessible exit options, and user control are complementary questions for platform evaluation. Privacy and asymmetric data power also remain relevant to responsible platform governance (Véliz, 2020). These issues provide design and research context rather than outcomes measured by the present model.

Evaluations of repeated engagement should therefore combine reported frequency with direct measures of automaticity, perceived control, and well-being.

## 5 Conclusion

This study examined a frequency-moderated UTAUT model of engagement with AI virtual-avatar livestreams and videos.

After excluding PE4, effort expectancy and social influence were positively associated with behavioral intention, while performance expectancy was not significant. Behavioral intention and facilitating conditions were positively associated with self-reported continued-use tendency. The two frequency interactions were significant, but the direct frequency path was not. These patterns support further examination of use frequency as a boundary condition in AI virtual-avatar engagement. Their interpretation remains exploratory given construct overlap and the sample and item revisions made after data collection.

First, the sample was recruited through online snowball channels, and the restriction to 165 adults with prior exposure was introduced *post hoc*. This restricts generalizability and may introduce selection bias. The 170 ‘almost never’ respondents had no scale responses in the available export, preventing a full 337-versus-165 structural sensitivity comparison. The record audit in Supplementary Table S4 documents this limitation rather than establishing that the exclusion had no effect. Future studies should prespecify eligibility criteria and retain the information needed to examine exclusions.

Second, the cross-sectional design cannot establish causal direction or within-person change. Longitudinal or experimental designs are required to test whether the relative roles of intention and facilitating conditions change as the same individuals accumulate experience.

Third, frequency was self-reported in four ordered monthly categories rather than obtained from platform logs. Treating the 1–4 coding as an approximately linear moderator imposes an equal-step approximation across unequal count ranges; future research should use continuous behavioral counts, platform logs, or preregistered ordinal-group comparisons. The outcome was also a self-reported continued-use tendency, not objective behavioral telemetry. Because predictors and outcomes came from the same questionnaire, common-method variance and consistency motives cannot be ruled out.

Fourth, removing the emotional-attachment item PE4 improved several PE-related HTMT values, but nine of 15 construct pairs remained above 0.90. The especially high BI–UB, SI–BI, and SI–UB values require cautious interpretation of separate construct effects. PE1–PE3 still emphasize avatar and interaction qualities rather than conventional task-performance benefits, SI3 combines social and attention content, and UB combines advocacy, prospective continuation, and perceived behavioral change. The revised item specification was evaluated on the same sample. Cognitive interviews, independently piloted item sets, and validation in a new sample would help distinguish these domains before testing structural relationships. Future designs should also separate intention measurement from later behavioral records.

The processes underlying the weaker BI-UB and stronger FC-UB associations at higher reported frequency remain unresolved. Plausible explanations include platform familiarity, stable differences between users, access conditions, and habit-related processes.

Longitudinal panels should repeatedly measure frequency, automaticity, perceived autonomy, and facilitating conditions, then connect these measures to behavioral logs. This design would test whether the between-person interaction pattern corresponds to within-person change.

Experimental studies could manipulate interface support or persuasive cues to test whether they alter the relative contributions of intention and facilitating conditions.

Research in ethically sensitive AI contexts has operationalized emotional vulnerability and ethical concern directly (Fu et al., 2025). Future virtual-avatar studies could likewise measure perceived control, privacy concern, cognitive offloading, and well-being alongside acceptance constructs.

Comparative work should also examine whether these relationships vary across age groups, cultural settings, and avatar roles, including AI anchors, virtual influencers, and virtual idols.

Together, these designs would help determine whether the observed moderation reflects exposure, automaticity, platform support, or other stable differences between users.

## Data Availability

De-identified data supporting the conclusions of this article are available from the corresponding author, Ge Wu (wuge1880@gdut.edu.cn), on reasonable request, subject to applicable ethics and participant-privacy requirements. The Supplementary material provides the complete HTMT outputs, additional measurement diagnostics, the sample-accounting audit, and the comparison of measurement specifications.
