# Supplementary material for: Acceptance and self-reported continued use of AI virtual avatars: an extended UTAUT model with the moderating role of use frequency
Source: Front Psychol. 2026 Oct 1;17:1957852. doi: 10.3389/fpsyg.2026.1957852 (PMC13669095; doi:10.3389/fpsyg.2026.1957852)
Supplement: Supplementary file 1 [file Data_Sheet_1.pdf]

Supplementary Material

Tables S1–S6 provide the complete discriminant-validity outputs, additional measurement diagnostics, sample accounting, and comparisons of the two measurement specifications. The final model excludes SI2 and PE4, retains EE1–EE4 and UB1–UB3, and uses 165 respondents.

Table S1. Complete 21-pair HTMT list and 95% percentile bootstrap confidence intervals.

Construct pair	HTMT	Bootstrap mean	2.5%	97.5%
EE–BI	0.895	0.896	0.814	0.967
FC–BI	0.923	0.924	0.836	1.008
FC–EE	0.819	0.820	0.726	0.905
Freq–BI	0.312	0.312	0.143	0.476
Freq–EE	0.169	0.175	0.044	0.333
Freq–FC	0.330	0.330	0.158	0.493
PE–BI	0.857	0.857	0.753	0.954
PE–EE	0.893	0.892	0.804	0.965
PE–FC	0.832	0.833	0.728	0.927
PE–Freq	0.194	0.197	0.044	0.368
SI–BI	0.977	0.979	0.897	1.056
SI–EE	0.942	0.943	0.878	0.998
SI–FC	0.935	0.938	0.858	1.012
SI–Freq	0.278	0.279	0.117	0.441
SI–PE	0.938	0.939	0.857	1.013
UB–BI	0.980	0.982	0.915	1.050
UB–EE	0.927	0.928	0.862	0.990
UB–FC	0.938	0.939	0.852	1.016
UB–Freq	0.301	0.302	0.129	0.463
UB–PE	0.850	0.849	0.746	0.943
UB–SI	0.968	0.969	0.895	1.035

Note. Estimates and intervals are reproduced from the final SmartPLS bootstrap report: 5,000 resamples, percentile intervals, two-tailed tests, fixed seed. The 15 pairs among PE, EE, SI, FC, BI, and UB are the multi-item discriminant-validity comparisons. Frequency is a single observed item; software-reported entries involving it are included for completeness and are not evidence about the validity of a multi-item frequency scale. The intervals are unadjusted pairwise intervals, not a familywise test of the complete model.

Table S2. Complete extended HTMT matrix from the algorithm export, including generated interactions.

	**BI**	**EE**	**FC**	**Freq**	**PE**	**SI**	**UB**	**F×FC**	**F×BI**
BI	—	—	—	—	—	—	—	—	—
EE	0.895	—	—	—	—	—	—	—	—
FC	0.923	0.819	—	—	—	—	—	—	—
Freq	0.312	0.169	0.330	—	—	—	—	—	—
PE	0.857	0.893	0.832	0.194	—	—	—	—	—
SI	0.977	0.942	0.935	0.278	0.938	—	—	—	—
UB	0.980	0.927	0.938	0.301	0.850	0.968	—	—	—
F×FC	0.129	0.158	0.160	0.135	0.125	0.129	0.160	—	—
F×BI	0.034	0.081	0.123	0.060	0.099	0.060	0.045	0.720	—

Note. F×FC = Frequency × Facilitating Conditions; F×BI = Frequency × Behavioral Intention. The software's matrix also contains the generated interaction variables, although its bootstrap HTMT list contains only the 21 pairs in Table S1. These generated variables are mathematical products, not additional psychological constructs; their matrix entries are included for output completeness. No bootstrap intervals have been invented for these additional entries.

Table S3. Fornell–Larcker comparisons in the final measurement model.

	**BI**	**EE**	**FC**	**Freq**	**PE**	**SI**	**UB**
BI	0.853	—	—	—	—	—	—
EE	0.761	0.857	—	—	—	—	—
FC	0.756	0.701	0.854	—	—	—	—
Freq	0.281	0.157	0.295	1.000	—	—	—
PE	0.707	0.765	0.690	0.177	0.866	—	—
SI	0.804	0.807	0.773	0.254	0.779	0.865	—
UB	0.793	0.781	0.766	0.269	0.696	0.791	0.847

Note. Diagonal entries are square roots of AVE; lower off-diagonal entries are construct-score correlations. Frequency has a single indicator. Meeting this comparison does not resolve the elevated HTMT values in Tables S1–S2.

Table S4. Audit of post-hoc sample restriction and feasibility of a full-sample comparison.

Record-accounting step	n	Available scale responses / interpretation
Original questionnaire export	337	All submitted records; not 337 complete scale profiles
"Almost never" frequency category	170	No responses to any of the 21 original scale items
Other frequency categories	167	Complete responses to all 21 original scale items
Additional complete records under 18	2	Excluded from the adult analytical population
Final analytical sample	165	Complete adult records; 19 indicators after removing SI2 and PE4

The 170 no-item-response records correspond exactly to frequency code 5 in the original export. Three of these 170 respondents were also under 18; they are already counted in that exclusion. Two further respondents under 18 had complete scale data. The 165 retained records match the original export row for row before the documented score transformation. Thus, sample restriction did not change retained item responses.

A 337-versus-165 structural sensitivity comparison cannot be estimated from the supplied export without assigning unobserved measurement profiles to the 170 respondents. This audit establishes record counts and data availability, not the absence of selection bias. The post-hoc restriction limits inference to the retained adult population. Tables S5–S6 instead evaluate item-specification sensitivity on the same 165 respondents and should not be interpreted as a substitute for a sample-selection sensitivity analysis.

Table S5. All 15 multi-item HTMT comparisons before and after PE4 deletion on the same sample.

Construct pair	PE4 retained	PE4 deleted	Change
PE–EE	0.928	0.893	-0.035
PE–SI	0.979	0.938	-0.041
PE–FC	0.906	0.832	-0.074
PE–BI	0.907	0.857	-0.050
PE–UB	0.901	0.850	-0.051
EE–SI	0.942	0.942	0.000
EE–FC	0.819	0.819	0.000
EE–BI	0.895	0.895	0.000
EE–UB	0.927	0.927	0.000
SI–FC	0.935	0.935	0.000
SI–BI	0.977	0.977	0.000
SI–UB	0.968	0.968	0.000
FC–BI	0.923	0.923	0.000
FC–UB	0.938	0.938	0.000
BI–UB	0.980	0.980	0.000

Note. Both specifications exclude SI2 and retain all four EE items and all three UB items ($n = 165$). Negative changes indicate lower HTMT. Thirteen ratios exceeded 0.90 with PE4 retained, compared with nine after deletion. Only comparisons involving PE change because HTMT is computed from indicator correlations. Values were independently checked against the supplied item data; a uniform $8 - x$ transformation does not change these absolute-correlation-based ratios.

Table S6. Structural-path sensitivity to PE4 deletion on the same sample.

Hypothesis/path	PE4 retained β	p	PE4 deleted β	p
H1: PE → BI	0.181	0.036	0.107	0.176
H2: EE → BI	0.250	0.006	0.280	0.002
H3: SI → BI	0.455	<0.001	0.495	<0.001
H4: FC → UB	0.397	<0.001	0.397	<0.001
H5: BI → UB	0.470	<0.001	0.470	<0.001
H6a: Freq × FC → UB	0.170	0.022	0.170	0.022
H6b: Freq × BI → UB	-0.178	0.015	-0.178	0.015
H6c: Freq → UB	0.031	0.491	0.031	0.491

Note. n = 165 in both specifications; SI2 was excluded in both, and EE1–EE4 and UB1–UB3 were retained. Estimates are from the corresponding SmartPLS bootstrap reports, each with 5,000 resamples. The PE–BI coefficient changed from statistically significant to nonsignificant. Both interaction signs and their significance decisions were unchanged. This documents measurement sensitivity; it does not establish confirmatory robustness to post-hoc respondent exclusion.

Data provenance: the final algorithm, bootstrapping, blindfolding, and PLSpredict exports all correspond to the PE4-deleted specification with 19 multi-item indicators. Tables S1–S3 and S6 contain software-exported estimates; Tables S4–S5 add the source-data audit and independently checked HTMT comparisons.
